# Solid Lipid Nanoparticles vs. Nanostructured Lipid Carriers: A Comparative Review

**DOI:** 10.3390/pharmaceutics15061593

**Published:** 2023-05-25

**Authors:** Cláudia Viegas, Ana B. Patrício, João M. Prata, Akhtar Nadhman, Pavan Kumar Chintamaneni, Pedro Fonte

**Affiliations:** 1 Center for Marine Sciences (CCMar), University of Algarve, Gambelas Campus, 8005-139 Faro, Portugal; 2 Faculty of Medicine and Biomedical Sciences (FMCB), University of Algarve, Gambelas Campus, 8005-139 Faro, Portugal; 3 iBB—Institute for Bioengineering and Biosciences, Department of Bioengineering, Instituto Superior Técnico, Universidade de Lisboa, 1049-001 Lisboa, Portugal; 4 Associate Laboratory i4HB—Institute for Health and Bioeconomy, Instituto Superior Técnico, Universidade de Lisboa, Av. Rovisco Pais, 1049-001 Lisboa, Portugal; 5 Institute of Integrative Biosciences, CECOS University, Hayatabad, Peshawar 25000, Pakistan; 6 Department of Pharmaceutics, GITAM School of Pharmacy, GITAM-Hyderabad Campus, Hyderabad 502329, Telangana, India; 7 Department of Chemistry and Pharmacy, Faculty of Sciences and Technology, University of Algarve, Gambelas Campus, 8005-139 Faro, Portugal

**Keywords:** lipid nanoparticle, NLC, nanocarrier, nanoparticle characterization, nanoparticle production, SLN

## Abstract

Solid–lipid nanoparticles and nanostructured lipid carriers are delivery systems for the delivery of drugs and other bioactives used in diagnosis, therapy, and treatment procedures. These nanocarriers may enhance the solubility and permeability of drugs, increase their bioavailability, and extend the residence time in the body, combining low toxicity with a targeted delivery. Nanostructured lipid carriers are the second generation of lipid nanoparticles differing from solid lipid nanoparticles in their composition matrix. The use of a liquid lipid together with a solid lipid in nanostructured lipid carrier allows it to load a higher amount of drug, enhance drug release properties, and increase its stability. Therefore, a direct comparison between solid lipid nanoparticles and nanostructured lipid carriers is needed. This review aims to describe solid lipid nanoparticles and nanostructured lipid carriers as drug delivery systems, comparing both, while systematically elucidating their production methodologies, physicochemical characterization, and in vitro and in vivo performance. In addition, the toxicity concerns of these systems are focused on.

## 1. Introduction

Nanomedicine aims to provide accurate diagnoses and treatments for diseases more effectively, with minimal adverse effects. Nanomedicine has gained popularity because of its efficiency in delivering drugs and other bioactives to target tissues more accurately in a controlled manner by encapsulating or attaching them to nanostructures [1,2]. These drug delivery systems involve nanocarriers that are colloidal drug carrier systems having submicron particle sizes, typically below 1000 nm. Due to their high surface area to volume ratio, nanocarriers can modify the basic properties and bioactivity of drugs. They also allow drug protection (e.g., from humidity, pH changes, and enzymes); improved pharmacokinetics and biodistribution of the drugs; either by passive or active targeting, resulting in reduced toxicities and improved therapeutic benefits [3,4,5]; enhanced bioavailability; controlled drug releasing profiles; prolonged blood circulation times; enhanced intracellular penetration; and site- and organ-specific targeted delivery. 

Different types of materials have been used to produce nanoparticles, mainly polymeric, lipid, and inorganic materials. Among these, lipid nanocarriers have considerable advantages due to their biocompatibility, biodegradability, low toxicity, scale-up capacity, and delivery of both hydrophilic and lipophilic drugs in a controlled or targeted manner [6,7]. These carriers may also permeate physiological barriers, such as the blood–brain barrier and the intestinal epithelium [8]. Further, to combine the advantages of different materials, hybrid nanoparticles may also be obtained to improve the features of lipid-based nanoparticles. For example, a new approach can be developed based on the physical modification of the lipid matrix with polymers, producing a lipid–polymeric matrix to entrap the drugs [9,10,11].

Intralipid^®^ was the first safe lipid parental emulsion developed in the 60s [12]. In the same decade, liposomes were first described by Bangham et al., and since then, liposomes have become the traditional models for lipid-based formulations [13,14]. A spherical vesicle with an aqueous nucleus enclosed by a lipidic bilayer membrane is what defines a liposome. Since then, liposomes have been extensively studied from pharmaceutical to cosmetic applications, due to their advantages as a carrier system with the additional benefit of protection from enzymatic activity. However, further development of liposomal formulations has been hindered by its major obstacles. Some of those are the limited physical stability of the liposomal suspension, drug leakage, low targeting ability, non-specific clearance by monocytes and macrophages, and up-scaling difficulties [3,15,16,17]. Several other carrier systems of a lipidic nature were developed, such as nanopellets for perioral administration, described by Speiser and colleagues (lipidic microparticles), nanosuspensions produced by ball milling or high-pressure homogenization (HPH), lipospheres [18,19,20], and many others.

With the introduction of liposomal Doxorubicin, Doxil^®^ entered into the United States market in 1995 for the treatment of AIDS-related Kaposi sarcoma and ovarian cancer, thereby increasing the interest in lipid-based drug delivery systems [14,21,22]. Further, scientists investigated new and advanced drug delivery technologies combining the advantages of liposomes, nanoemulsions, and solid lipid nanoparticles (SLN) [18]. SLNs are solid colloidal particles in the sub-micron range, ranging from 4 to 1000 nm, and contain in their formulation physiological, biodegradable, and biocompatible lipids and surfactants, which can incorporate both lipophilic and hydrophilic drugs inside the lipid matrix. The solid lipid is the dispersed phase, whereas the surfactant acts as an emulsifier. The first one is usually produced from triglycerides, glyceride mixtures, or even waxes, and it remains in the solid state at room and body temperature. On the other hand, to enhance formulation stability, the range of surfactant concentration is typically 0.5 to 5% (*w*/*v*). The choice of lipids and surfactants greatly impacts the physicochemical properties (e.g., particle size and drug loading) of the nanoparticle formulation [3,17]. In comparison with liposomes, SLNs have better drug stability and prolonged release, and in comparison with polymeric nanoparticles, they are safer due to the absence of organic solvents in the production phase. More recently, nanostructured lipid carriers (NLCs) have been developed by adding a liquid lipid to the matrix, enhancing the advantages of SLNs [15]. For that reason, NLCs are usually denominated as second-generation lipid nanoparticles. In fact, NLCs have improved stability and drug loading capacity, preventing unwanted drug expulsion during shelf life. They are distinguishable from SLNs due to their solid matrix composition, where the lipidic phase contains both solid and liquid lipids at the body and room temperature [23]. 

Overall, this review aims to perform a comparison between SLNs and NLCs, discussing their advantages and disadvantages. The production methods to obtain them are briefly discussed, and more importantly, their in vitro and in vivo performance are scrutinized. Some toxicity concerns are addressed. 

## 2. Structural Features of Solid Lipid Nanoparticles and Nanostructured Lipid Carriers

**Figure 1:**
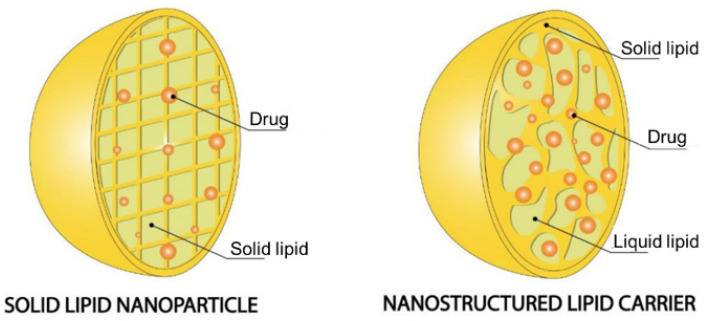
Structural matrix of SLN and NLC. Adapted with permission from [25].

Lipid particles with sizes on a nanoscale possess unique physical, mechanical, chemical, and biological features, which may differ greatly from their core components in SLNs and NLCs (Figure 1). These nanostructures are essentially composed of lipids and surfactants and can be used to deliver therapeutic agents (by encapsulation, incorporation, or/and surface attachment) and deliver them to target tissues [2,24]. Table 1 describes the compounds mostly used in their production. The structural key feature differences between SLNs and NLCs are represented in the following sections.

### 2.1. Solid Lipid Nanoparticles

SLNs were first described by Müller and Lucks in the early 90s, alongside the description of the method used for production in a patent application [26]. However, the term SLN was only used a few years later by the same group, when describing a method for loading magnetite into nanoparticles [18]. SLNs are composed of a physiological lipid that is solid at room and body temperature, a surfactant, and water [15,17,27]. The SLN lipidic phase is usually produced from steroids, di- or triglycerides, glyceride mixtures, or even waxes, typically used at 0.1 and 30% (*w*/*v*) and remaining in the solid state at room and body temperature. On the other hand, the surfactant concentration is in a range of 0.5 to 5% (*w*/*v*), in the generally recognized as a safe (GRAS) category [17,27,28,29]. A combination of surfactants can be also used to improve the stability of the SLN [30]. 

**Figure 2:**
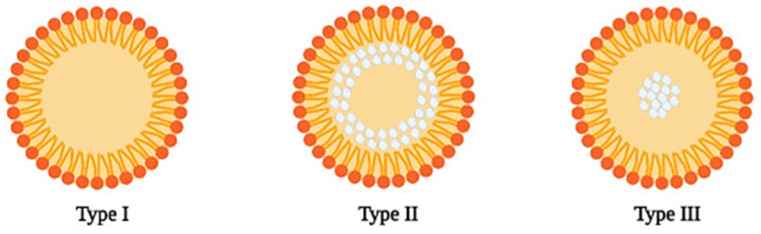
Structure of the 3 different types of SLN: homogenous matrix model (Type I), drug-enriched shell model (Type II), and drug-enriched core model (Type III). The drug is represented in white color. Reprinted with permission from [35].

The structure of SLNs depends on various factors, such as the components of the formulation, the solubility of the compounds including the drug, and the production method. Three different structures of SLN are reported [15,31]. In the SLN homogeneous matrix model (Type I) (Figure 2), the particles are produced by a cold or hot homogenization technique for very lipophilic drugs. In the first method, the drug is dissolved in a lipid matrix, and, due to high-pressure homogenization, mechanical breakings cause nanoparticle formation. In the second method, the lipid is dissolved in a lipid matrix while increasing the temperature and the nanoparticles are formed similarly. In the SLN drug-enriched shell model (Type II) (Figure 2), the particles may be produced by the hot homogenization technique. During the cooling of the system, the lipid molecules precipitate first, forming a lipid core. Meanwhile, the concentration of the drug increases in the rest of the melted lipid until its solubility limit is reached. When this point is reached, the mix of drug and melted lipid crystalizes, forming an outer shell. This model is not the best for prolonged release of the drug but can be very interesting to increase drug penetration with topical application, especially when associated with the occlusive effect of SLNs. Lastly, the SLN drug-enriched core model (Type III) (Figure 2) forms nanoparticles when the drug concentration is close to its solubility limit in the melted lipid. This type is the opposite of type II since the first compound to precipitate is the drug, which forms the core, and the shell is composed of the lipid and a low concentration of the drug [15,29,31,32,33,34]. Table 2 describes several examples of studies about the development, applications, and characterization of SLNs.

In addition to the advantages, SLNs also have relevant disadvantages such as possible aggregation, instability during storage, and low drug loading for some drugs [7]. For these reasons, and to overcome them, NLCs have been developed.

### 2.2. Nanostructured Lipid Carriers

NLCs are the second generation of lipid nanocarriers and were created in the late 90s to solve the disadvantages of SLN [15,31]. NLCs are composed of a mixture of solid and liquid lipids, in a ratio of up to 70:30, respectively, and an aqueous phase composed of a surfactant [45,46]. The lipids used in these formulations are biologically compatible, which is important to reduce toxicity [47].

**Figure 3:**
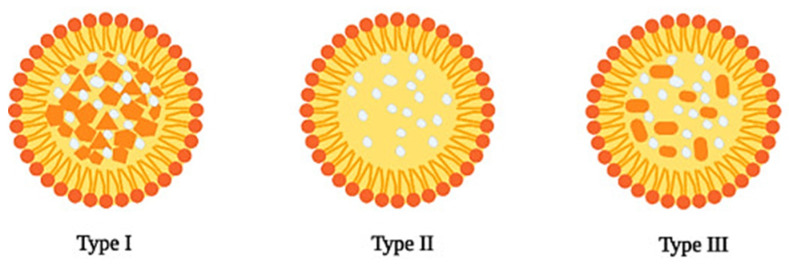
Structures of different types of NLC: imperfect crystal (Type I), amorphous (Type II), and multiple type (Type III). The drug is represented in white. Reprinted with permission from [35].

Incorporating both liquid lipids and solid lipids results in a more imperfect matrix in the NLC structure, thus resulting in more efficient drug loading and incorporation [48]. NLCs can be classified, according to their structure, into three different types (Figure 3), which differ mainly in their lipid composition.

The imperfect crystal type (Type I) results from mixing lipids with different chain lengths or by using either mono-, di- or triglycerides. By doing so, a matrix comprising several voids and imperfections provides a more suitable environment for drug incorporation. The amorphous type (Type II) is obtained by using medium chain length triglycerides along with solid lipids. The solid lipids do not undergo recrystallization after NLC cooling, thus resulting in solid particles with an amorphous structure. By not recrystallizing during the cooling phase, and even during storage, the unwanted release of the drug is reduced, thus improving its shelf life. Multiple type NLCs (Type III) are obtained by mixing solid lipids with an oil such as an oleic acid and/or medium and long-chain triacylglycerols. To achieve a multiple type NLC, the oil must be mixed in a ratio above its solubility in the solid lipid, resulting in the formation of very small oil compartments (nanocompartments) in the NLC matrix during the cooling phase of the nanoemulsion [31,32,48]. Table 3 summarizes several examples of studies about the development and application as well nanoparticle characterization of NLC.

### 2.3. Comparison between Solid Lipid Nanoparticles and Nanostructured Lipid Carrier

SLNs have been widely studied as systems for drug delivery for several delivery routes, such as oral, parenteral, and topical delivery. Due to their structure, which can be finely tuned depending on the chemical profile of its active ingredients and excipients, one expects several advantages. However, the modified release property is dependent on the solid state of the particle, e.g., crystallization and physicochemical transitions. Retarded or suppressed crystallization had been reported, resulting in a poor drug payload. Lipidic materials typically undergo physicochemical transitions, e.g., matrix molecule rearrangement. This phenomenon results in not only a more densely packed and arranged matrix, but also an altered shape, which presents an unfavorable localization for many drug molecules. The payload depends not only on the physicochemical properties of the drugs to be incorporated but also on the type of matrix material. Drugs that could not be incorporated inside the SLN matrix may adsorb on the nanoparticle surface or even lead to the separation of the particle matrix [3,7,9,32,59]. To overcome unwanted drug release during storage, a more imperfect matrix is desirable. To accomplish that, the use of spatially different molecules is needed. 

NLCs are formed by mixing different combinations of solid and liquid lipids, in a ratio from 70:30 to 99:1, which remain in the solid state at both body and room temperature, and by controlling the content of liquid lipids in the formulation, improved incorporation and immobilization of drug molecules is achieved. Whereas in SLNs, the drug molecules are dispersed in their molecular form, in NLCs the imperfections in the matrix formed by the differences between solid and liquid lipids leads to more spaces available for drugs, which improves the incorporation of drug molecules in both molecular form and amorphous clusters, also avoiding the potential expulsion of the active compound during storage [3,9,32,59,60,61]. In Table 4 their classification by properties is shown, which highlights the comparison between SLNs and NLCs. 

## 3. Production Methods of Solid–lipid Nanoparticles and Nanostructured Lipid Carriers

**Figure 4:**
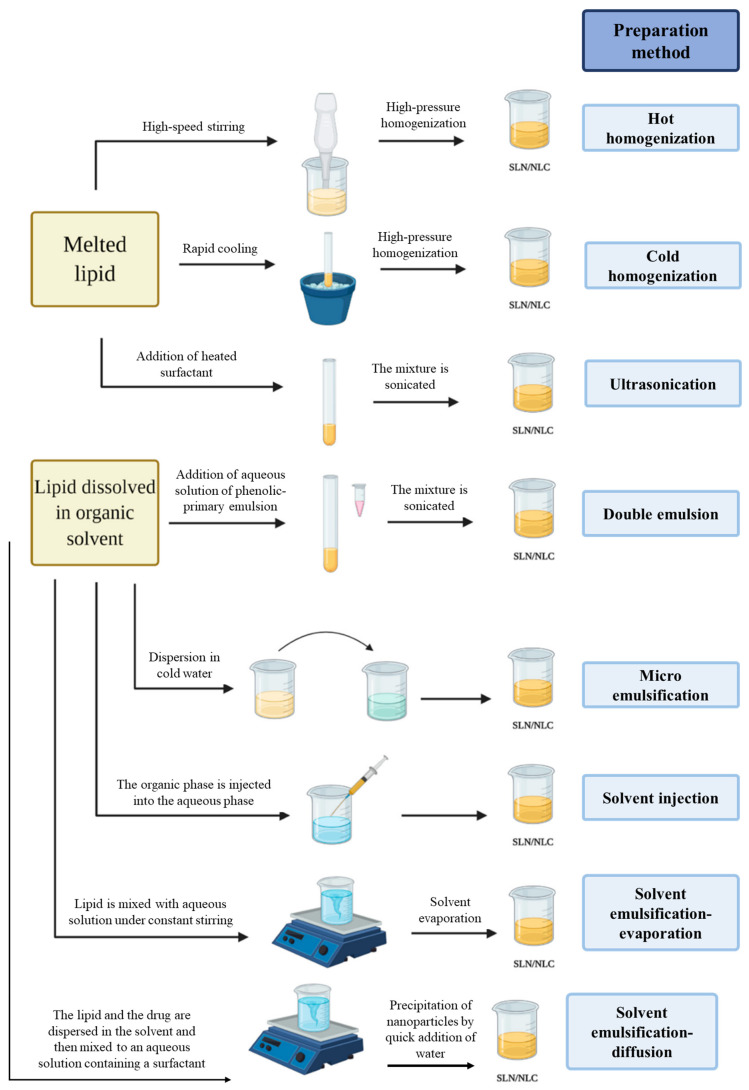
Production methods to obtain SLNs and NLCs. Adapted from [35].

**Figure 5:**
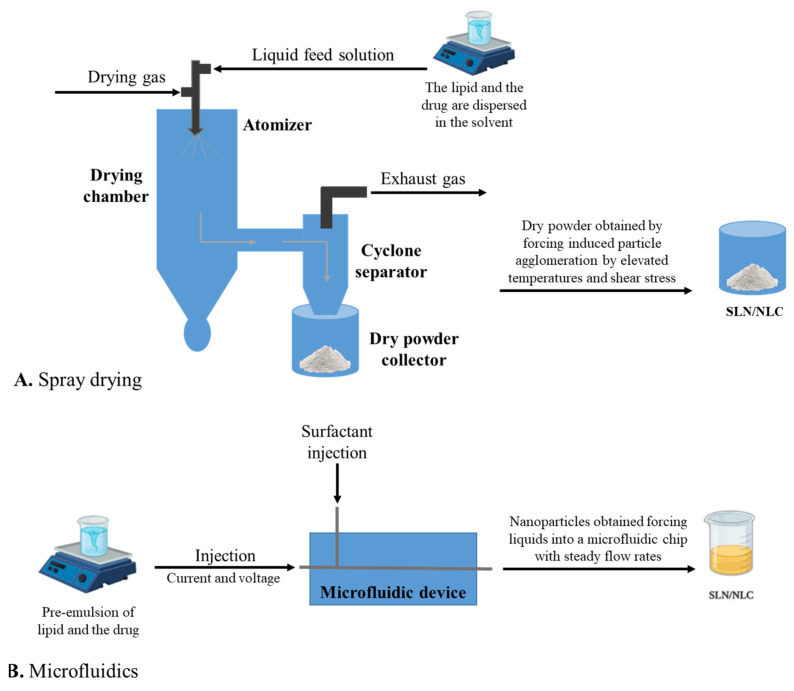
Spray drying (**A**) and microfluidics (**B**) methods to obtain SLN and NLC.

There are several methodologies to produce lipid nanoparticles such as SLNs and NLCs. The most common methods are high-pressure homogenization (hot and cold), microemulsification, solvent emulsification (evaporation or diffusion), solvent injection, double emulsion, ultra-sonication or high-speed homogenization, spray drying, and microfluidics. These methods are schematized in Figure 4 and Figure 5 and are briefly described in the following sections.

### 3.1. High-Pressure Homogenization

HPH is the most common method and consists of a potent and trustworthy technique for the preparation of SLNs and NLCs [62]. There are two types of high-pressure homogenization, hot HPH, and cold HPH. In both, the first step is to dissolve the drug in a solid lipid melted at approximately 5–10 °C above its melting point [27,63].

Hot HPH consists of adding an aqueous surfactant solution at the same temperature as the melted lipid to the drug–lipid melt. After this step, the substances are homogeneously dispersed by high-shear mixing, forming the pre-emulsion. The hot pre-emulsion is then submitted, at the same temperature, to high-pressure homogenization to reduce particle size to the nanoscale. Generally, three cycles at 500 bars are appropriate. The last step is cooling down the oil-in-water (*o*/*w*) nanoemulsion to room temperature, which leads to lipid re-crystallization, thus forming the solid matrix of the lipid nanoparticle [27,64]. 

The cold HPH method consists of rapidly cooling the drug–lipid melt, using either dry ice or liquid nitrogen. The rapid cooling forces the drug to be homogenously mixed within the lipid matrix. Afterward, the obtained solid is pulverized into particles within the micron range (with either a ball mill or mortar), followed by its dispersion in an aqueous surfactant solution which is already cooled down (pre-emulsion). The pre-emulsion is then subjected to high-pressure homogenization at room or below room temperature, breaking the microparticles into nanoparticles [27,62,64].

### 3.2. Ultra-Sonication or High-Speed Homogenization

Ultra-sonication and high-speed homogenization are used to mix the lipid phase and the containing surfactant aqueous phase [65,66]. Nanoparticles obtained by this method usually have high polydispersity, which can be dealt with by using probe-based sonicators. Despite obtaining a less dispersed distribution, there is a risk of cross-contamination from the probe metal [67]. Even if this method has its drawbacks, high-speed homogenization, for example, becomes much more efficient, simpler, cheaper, and easier to reproduce if multiple cycles of homogenization at high velocities and high pressure (100–200 MPa) conditions are implemented. 

### 3.3. Double Emulsion

The double emulsion method involves the preparation of a primary emulsion (*w*/*o*), which consists of dissolving a drug molecule (usually hydrophilic) in an aqueous solvent (inner aqueous phase) that is dispersed in a lipid phase containing an emulsifier, known as the oil phase. Afterward, an aqueous solution containing a hydrophilic emulsifier is added to the primary emulsion, which forms a double emulsion (*w*/*o*/*w*) after stirring. Since this method generates nanoparticles with high polydispersity, it is not recommended for delivery in some administration routes [27,68,69].

### 3.4. Microemulsion Method

The microemulsion method was developed by Gasco et al., and similarly to HPH, it starts by melting the solid lipid(s) at a temperature higher than its/their melting points (by 5 to 10 °C) and dissolving the drug in melted lipid(s) [70]. In the following step, an aqueous surfactant solution with a temperature above the temperature of the melted lipid is added to the drug–lipid melt with continuous stirring until a transparent microemulsion is obtained. The microemulsion formed is dispersed in cold water by gentle stirring and the microparticles are broken into nanoparticles which crystallize to form the SLN or NLC. Nanoparticles produced by this method are diluted, so at the end of the process, the preparation needs to be concentrated by ultrafiltration or lyophilization. The main disadvantage is the need for a high concentration of surfactants [64,71]. 

### 3.5. Solvent Injection

This novel approach consists of lipids being dissolved in water-miscible organic solvents pharmacologically accepted, such as ethanol, acetone, or isopropanol, followed by injection in an aqueous phase with constant mixing, causing the lipid precipitation. The dispersion is then filtered to remove excess lipid content. By adding an emulsifier to the aqueous phase, lipid droplets form at the injection site and stabilize the particle until solvent diffusion occurs [72].

### 3.6. Solvent Emulsification-Evaporation

In this method, the solid lipid is dissolved in an organic solvent and afterward emulsified in an aqueous solution while stirring. The organic solvent evaporates during stirring, forming nanoparticles by precipitation of the lipid in the aqueous phase. The concentration of the lipid will directly impact the size of the particles. This method is suitable for thermolabile drugs due to the absence of thermal stress. However, this method is not suitable for drugs capable of interacting with the organic solvent [64,73].

### 3.7. Solvent Emulsification-Diffusion

The solvent emulsification-diffusion technique uses a partially water-miscible organic solvent containing saturated water to achieve thermodynamic equilibrium, avoiding the diffusion of the solvent from the droplets into the aqueous phase. The lipid and the drug are dispersed in the solvent and then added to an aqueous solution containing a surfactant, forming an *o*/*w* emulsion. The particles are formed by adding more water, which facilitates solvent diffusion into the continuous phase and incites precipitation of the nanoparticles [74,75].

### 3.8. Spray Drying

Spray drying is a common method for high melting point lipids. It is also used as an alternative method to lyophilization in SLN and NLC formulations [76]. The principle behind spray drying consists of inducing particle agglomeration through elevated temperatures and shear stress, resulting in partial melting and increased kinetic energy. This leads to multiple particle collisions. Despite being more efficient than other methods, it is rarely used due to the risk of inducing particle aggregation and structural changes in the lipid core and surfactant films or even particle degradation by high temperatures [77,78,79,80,81].

### 3.9. Microfluidics

Microfluidics is a more recent method, which has been introduced as a novel methodology to produce nanoparticles with optimized uniformity [82,83]. By forcing liquids into a microfluidic chip at steady flow rates, the nanoliter amount of these reagents collides and rapidly mixes under precise pressure [84]. Submitting a pre-emulsion to high-pressure microfluidics and cooling it down to room temperature can minimize the particles’ polydispersity, reduce production times, and avoid organic solvents. Overall, it could be a promising approach for the large-scale production of drug-loaded SLNs and NLCs [23,85].

## 4. In Vitro Performance of Solid Lipid Nanoparticles and Nanostructured Lipid Carrier

These lipid-based nanoparticles have potential as novel delivery system. Therefore, numerous studies have been carried out to evaluate the stability of these drug-loaded nanoparticles, their size, zeta potential, and entrapment efficiency, as well as in vitro studies to analyze their release profiles and bioavailability.

Previously, Yuan et al. highlighted the importance of conjugation by designing folic acid (FA)-modified paclitaxel-loaded SLNs. Their results showed an increase in the uptake of FA-SLN via folate receptor-mediated uptake in A549 cell lines. The paclitaxel was loaded in an FA-modified SLN, so the cytotoxicity of this drug was significantly enhanced because of the improved endocytosis mediated by the folate receptor compared with the free drug in solution [86]. 

In another study, Das et al. obtained different results when comparing SLNs and NLCs as delivery systems for clotrimazole. When comparing SLNs and NLCs with the same solid lipid and surfactant, statistical differences between the two were found in terms of particle size, zeta potential, polydispersity index (PdI), and encapsulation efficiency. In addition, for the same drug concentration, the particle size of the NLC was slightly inferior. They observed that drug release was slightly faster for the NLC at 80 h (56.6%) when compared to the SLN (42.5%). However, the differences between the drug release profiles of the two types of nanoparticles were not considered statistically relevant. Regarding stability, the NLC showed slightly better results than the SLN, especially at high drug loading [64]. Later, Dudiphala et al. compared SLN and NLC delivery systems of nisoldipine by characterizing them and analyzing their in vitro release and stability profile. They concluded that an NLC formed by oleic acid and dynasan-114 showed the best features regarding particle size, PdI, zeta potential, entrapment efficiency, and in vitro control release when compared to the optimal SLN formulation. The formulated NLCs were also stable for 3 months at room temperature after being lyophilized, supporting the advantages of the NLC in contrast with the SLN [16]. In contrast, Under et al., in an ex vivo study, found similar results from both SLNs and NLCs in the corneal delivery of loteprednol etabonate, a topical corticosteroid, when used in inflammatory and allergic conditions of the eye. Moreover, these lipidic nanoparticles formulation allow a more effective treatment, allowing no water loss by blockage of the pores in the corneal epithelium (especially dry eye disease) [87].

**Figure 6:**
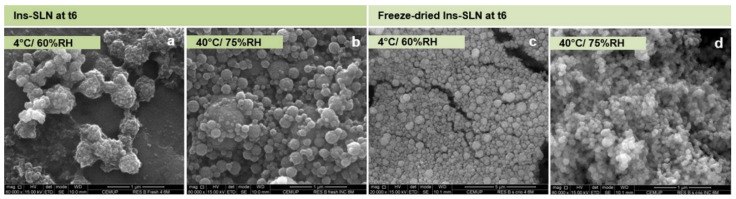
SEM microphotographs of insulin-loaded SLNs (Ins-SLN) stored at 4 °C/60% RH and 40 °C/75% RH after 6 months (t6). Scale bar: (**a**,**b**,**d**) 1 μm; (**c**) 5 μm. Reprinted with permission from [88].

To evaluate the effect of SLN protection on the stability structure of encapsulated proteins under the effects of several processing conditions, Soares et al. evaluated the physicochemical properties of an insulin-loaded SLN. This study emphasized the evaluation of insulin secondary structure after its encapsulation into SLNs after freeze drying using different cryoprotectants, and over 6 months under varying storage conditions of temperature and humidity. The insulin-loaded SLN had a mean particle size of about 400 nm, a zeta potential of about −13 mV, and an insulin encapsulation efficiency of 84%. It was found that after freeze drying, the SLN physicochemical properties and encapsulated insulin structure were maintained to a good extent (similarity around 92% and 84%, after production and freeze drying, respectively, comparatively to insulin native structure). In addition, after 6 months, freeze-dried insulin-loaded SLN, even without cryoprotectant, stored at 4 °C and 60% relative humidity (RH) and 40 °C and 75% RH (40 °C/75% RH) showed an identical degree of morphology with a well-defined and characteristic spherical shape (Figure 6). These results showed the robustness of the delivery system. Overall, this study demonstrated the ability of SLN to retain therapeutic protein structure after production, freeze drying, and upon storage [88]. The advantage of SLN in protecting proteins from enzymatic degradation in the gastrointestinal tract and enhanced uptake has been widely described in different studies regarding the oral delivery of insulin for the treatment of diabetes mellitus [17,89,90].

Another study with SLNs formulated by acoustic cavitation-assisted hot melt mixing was performed to evaluate the stabilizers’ effect on their physicochemical properties [91]. To prepare the SLNs, a lipid Compritol^®^ 888 ATO was used as a model lipid, the poorly water-soluble drug ketoprofen as a drug, and Gelucire 50/13, Poloxamer 407, and Pluronic F-127 were used as surfactants. The SLNs showed an encapsulation efficiency of approximately 90% and a drug loading efficiency of 12%. The size was up to 250 nm; however, with the increase of the surfactant concentration, a decrease in particle size occurred. The SLNs showed overall good stability in water up to 50 °C. The in vitro drug release over 72 h was more than 90% and increased with pH. In addition, a nontoxic effect was found in Raw 264.7 cells for each of the surfactants evaluated. Therefore, all the surfactants were considered adequate for the preparation of SLN with high encapsulation efficiency, drug loading, stability, and good biocompatibility.

In another study, Ban et al. modulated the bioavailability of curcumin, a compound that acts as a potent anti-inflammatory, which has low oral bioavailability due to its low solubility, hindering its use in therapies. They controlled the interfacial properties of the SLN using tristearin and emulsifiers in polyethylene glycol (PEG) mixtures, prepared by an oil-in-water technique, to improve the bioavailability of curcumin. These SLNs were produced to be administrated by the oral route, and most of the curcumin was encapsulated. These SLNs were processed in a simulated gastrointestinal environment, resulting in their digestion and emulsification. Then, after being digested and emulsified in the in vitro simulated gastrointestinal environment, mixed micellar curcumin permeability studies were conducted using mucus-covered Caco-2 monolayers. It was also showed that conjugation of curcumin encapsulated in SLNs with PEG promoted rapid permeation of the small intestinal epithelium due to the neutral surface charge of the micelles formed, which resulted in a 12.0-fold increase in bioavailability compared to the curcumin solution when used in a rat model. The bioavailability of curcumin in SLN was around 92–95%, meaning that most of the drug was soluble in the micellar fraction after digestion in the gastrointestinal tract, which is a significant improvement when compared with other delivery techniques [92]. This effect of conjugating SLNs with PEG, a hydrophilic polysaccharide, to avoid phagocytosis by macrophages, has already been previously described and it was also found that it promotes a sustained release and uptake through the intestinal epithelium [17,93].

Besides the therapeutical potential of lipid nanoparticles, these particles also have numerous applications in the food and cosmetic sector [25,94]. For instance, in the food industry, the research for new delivery systems that can mask the undesired flavor of phenolics, allow controlled release in the gastrointestinal tract and protection against degradation, and increase in solubility, led to the use of lipids as the best choice for producing nanoparticles [25,95]. Aditya et al. performed an in vitro study, comparing SLNs and NLCs for quercetin delivery. The stomach and intestinal conditions were simulated, and the nanoparticles were analyzed regarding quercetin bioaccessibility and release in intestinal conditions. Both SLNs and NLCs were stable under stomach conditions for 2 h, and both types of nanoparticles showed a 6-fold increase in size during that time, meaning the intestinal enzyme complex hydrolyzed them. However, when comparing these delivery systems in terms of quercetin bioaccessibility and release in intestinal conditions, NLCs showed superior results after 2 h. The SLNs and NLCs had a similar percentage of quercetin bioaccessibility in the first 30 min, 31%, and 36.7%. However, after the 2 h digestion, the NLCs demonstrated a bioaccessibility of 52.7% whereas accessibility in the SLNs was only 39.7%. These can be explained by the fact that the lipids on SLNs are arranged more densely and tightly, which can reduce the hydrolysis rate and capacity of the intestine. Despite all this, SLNs can still have a prominent role in controlled lipid digestion. Regarding the quercetin released under intestinal conditions, NLCs, due to the fact they have a more disorganized lipidic matrix, showed a superior result (79%) when compared to SLN (53%) [96]. Gonçalves et al. compared curcumin-loaded in solid lipid nanoparticles and nanostructured lipid carriers to evaluate curcumin in vitro release kinetics and nanoparticle stability. The nanosystems were also included in a model beverage to evaluate, by an in vitro digestion process, their physicochemical properties during the storage period. In the end, SLNs and NLCs showed good particle stability during storage and did not show an effect on stability concerning pH. In relation to the color the beverage with NLC showed slightly better stability revealing a loss of stability of curcumin in SLN, however, the beverage with SLN showed better curcumin bioaccessibility in comparison with the beverage with NLC, which reveals a high nutrient release when loaded in SLN [97]. Overall, the results of these studies can be useful for the development of new functional foods reinforced with lipid-based nanoparticles; however, the evaluation of sensorial characteristics should be further studied. 

The NLC liquid and solid lipid composition have a decisive impact on their stability. Babazadeh et al. designed a set of assays with diverse types of solid lipids, liquid lipids, and surfactants to produce medicinal–functional foods fortified by lipophilic nutraceuticals. It was found that the best formulation was composed of 15% of liquid lipid and 85% of solid lipid with a ratio of 6% of surfactant to emulsion [98].

Lipid nanoparticles are potential carriers of lipophilic bioactive compounds. In 2015, Madureira et al. developed SLNs for the delivery of rosmarinic acid incorporated into food matrices using carnauba wax as a lipidic matrix. Polysorbate 80 was used as a surfactant at concentrations of 1, 2, and 3% (*v*/*v*) and carnauba wax at 0.5, 1, and 1.5% (*w*/*v*) to evaluate physicochemical properties, surface morphology, and drug association efficiency. The SLNs measured between 35 and 927 nm and zeta potential between −38 and −40 mV, reflecting its colloidal stability due to the electric repulsion of SLNs. Additionally, reducing carnauba wax concentrations led to small-sized particles; however, the surfactant concentrations must be higher than 2% (*v*/*v*), because they reduce the interfacial tension in particle surfaces preventing their aggregation. High association efficiencies (>99%) were also found. In addition, at the time of production and after 28 days in refrigerated storage, these properties were maintained and no rosmarinic acid was released by the particles, which revealed a stable SLN structure and compatibility between rosmarinic acid and the waxy core [99]. 

In another study, Park et al. showed that NLCs improved the release profile of vitamin D3, a lipophilic vitamin, in gastrointestinal fluids. Moreover, the vitamin D3-loaded NLC proved to be stable over 20 days of storage at 25 °C under a wide range of pH levels. Therefore, the use of NLCs is a promising strategy for increasing the oral bioavailability of this vitamin [100]. Further, He et al. developed SLNs with an equal mixture of propylene glycol monopalmitate and glyceryl monostearate for the delivery of carvacrol as a model of lipophilic antimicrobial drug, obtaining a stable system for the potential for food and cosmetic applications [101]. Later, Li et al. showed the benefit of using NLCs as an efficient and stable delivery system for improving the stability of essential oils, which allowed them to overcome disadvantages such as chemical instability, low water solubility, and bioavailability [102]. 

The use of food degree products as lipid matrices is indeed beneficial as a delivery system safety. Shtay et al. developed and characterized an SLN formulation made with cocoa butter as a lipid core and a mixture of sodium stearoyl-2-lactylate and mono- and diglycerides of fatty acids as a surfactant blend for potential application in foods. They evaluated its stability after three months of storage, and it was found to have good storage stability over time, revealing the stability of its structure to be applied as a food delivery system [103]. 

Later, Hashemi et al. studied the use of NLCs as antioxidant nanocarriers for a conjugated linoleic acid (CLA) in pasteurized milk for food fortification. The NLCs had a mean particle size of 77 nm, a zeta potential of −12.3 mV, and an encapsulation efficiency of 98.2%. Regarding stability, Hashemi and her colleagues did not find a significant particle size increase after 40 days of storage at 22 °C and 4 °C. Furthermore, when comparing the NLC samples with the controls, there was a significant decrease in secondary oxidation products in the former. This proved the efficiency of NLCs in protecting CLA from environmental conditions [104]. More recently, Gonçalves et al. also compared SLNs and NLCs as carriers for curcumin delivery. This lipophilic compound also attracts researchers’ attention as an antioxidant in beverages and functional foods. After analyzing the stability, and bioaccessibility of curcumin after in vitro digestion, NLCs displayed better stability and curcumin bioavailability when compared to SLN [105]. 

As it was mentioned before, both SLNs and NLCs can also have a significant role in the cosmetic industry [106]. It has been reported that NLCs can improve skin hydration and drug penetration into the skin. Several studies have tried to enhance dermal delivery with NLCs by decreasing their particle size or increasing their oil content. In 2014, Keck et al. developed ultra-small NLCs, with an average particle size of 85 nm, to encapsulate coenzyme Q10, and compared its in vitro release with typical NLCs and nanoemulsions. It was concluded that all delivery systems showed an efficient encapsulation, good PdI, and zeta potential. However, regarding the in vitro release profile, the ultra-small NLC presented a higher release of coenzyme Q10 compared to the other carriers, showing the potential of these novel carriers [107]. 

Another application that has been studied and can be a promising strategy for onychomycosis treatment is the use of SLNs and NLCs for topical administration of antifungal drugs, since in these therapies occlusive formulations are desired and intended to protect the drug from degradation. Thus, their hydrophobic nature allows them to form an occlusive film on the nail surface, which could also facilitate its diffusion across the nail plate and penetration. Lastly, these nanocarriers can accumulate in skin appendages or even allow control of their release through keratinized structures [108]. However, further research must be performed to overcome the challenges of nail antifungal delivery.

## 5. In Vivo Performance of Solid Lipid Nanoparticles and Nanostructured Lipid Carrier 

Different animal models have been used to evaluate the in vivo behavior of SLNs and NLCs. This section addresses some in vivo applications of these nanocarriers.

For most brain disorders, there is a lack of effective treatments available that can tackle those diseases due to the difficulty of the drug reaching the brain, which is protected by the blood–brain barrier (BBB) and the blood–cerebrospinal fluid barrier. The lipid nanoparticles have been studied as novel delivery systems that can cross the BBB and deliver the drug to its specific target [109]. Hsu et al. developed a cetyl palmitate-based, squalene cationic NLC, with PEG, Tween^®^ 80, and Pluronic^®^ F68 as interfacial excipients, and apomorphine as a model drug to assess BBB permeability. Cationic NLC formulations were compared with SLN and lipid emulsions, and the first ones showed a particle size of 370–430 nm, a zeta potential of 42–50 mV, and an encapsulation efficiency > 60%. The nanoparticle formulations also displayed slower release when compared to the other formulations. More importantly, in vivo bioluminescence studies of live rats showed the ability of NLCs to improve brain-specific delivery via certain blood vessels to selected regions of the brain. Therefore, NLCs were able to surpass the BBB and deliver drugs to the brain. This is due to the binding of positively charged NLCs to the paracellular area in the BBB, characterized to be rich in anionic sites. Thus, charged NLCs proved to be a success and a step forward in neurological disorders therapies [110].

**Figure 7:**
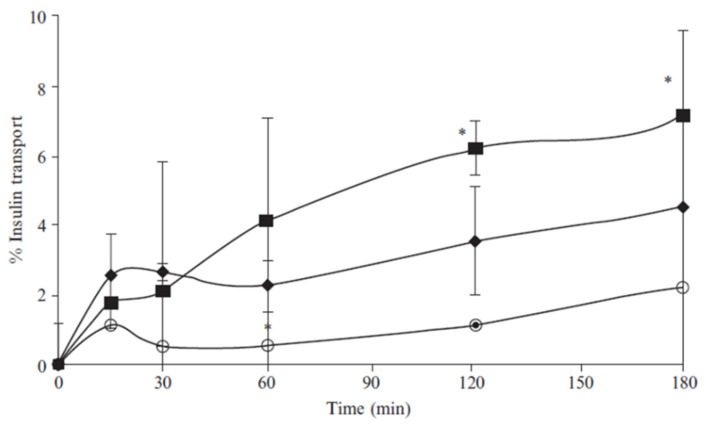
Cumulative permeation of insulin through Caco-2/HT29 co-culture monolayer loaded into SLN (filled diamonds), and into chitosan-coated SLN (filled squares), compared to insulin solution (circles). n = 3; mean ± SD; (*) *p* < 0.05, chitosan-coated SLN presented a significant difference from insulin solution. Reprinted with permission from [115].

**Figure 8:**
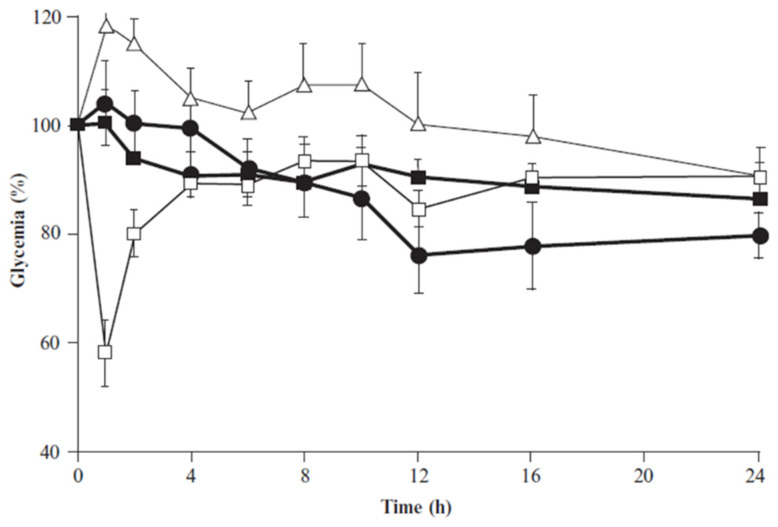
Decrease of plasma glucose concentration (%) in diabetic rats upon administration of subcutaneous insulin, 2.5 UI/kg (empty squares); oral insulin solution, 25 IU/kg (empty triangles); insulin-loaded SLNs, 25 IU/kg (filled squares); and insulin-loaded chitosan-coated SLN, 25 IU/Kg (filled circles). N = 6; mean ± SEM. Reprinted with permission from [115].

With the growing studying of nanotechnology and the possibility of modifying the surface of both SLNs and NLCs, novel studies have been developed to overcome the lack of specific target delivery. For example, in CRISPR/CAS-based gene editing and mRNA-based gene replacement techniques, it is crucial to have a delivery system that can target a specific organ or tissue to treat genetic problems using these new therapies [111]. Chitosan nanocarriers have been noted as a promising system for the delivery of therapeutic proteins or antioxidants. It is a positively charged polysaccharide acquired by partial deacetylation of chitin being biocompatible, biodegradable, and presenting mucoadhesive properties improving the permeation effect of hydrophilic compounds across epithelial barriers by opening tight intercellular junctions [112,113,114]. In 2011, Fonte et al. developed chitosan-coated SLNs as an interesting platform for the delivery of orally intended insulin to be used in the management of diabetes. Witepsol 85E was used as a lipid matrix and chitosan was adsorbed to SLN surfaces to improve insulin absorption in the gastrointestinal tract. Chitosan-coated SLN particles showed a particle size of about 450 nm and were positively charged, contrary to uncoated SLNs which were negatively charged. This feature takes the benefit of chitosan-coated SLN mucoadhesive and absorption-enhancing properties in Caco-2 intestinal cell monolayers and in the mucus-producing HT29 cells model. This was proved because chitosan-coated SLN showed higher cumulative transport of insulin across Caco-2 and Caco-2/HT29 co-culture monolayers compared to insulin solution (Figure 7). In addition, a more pronounced hypoglycemic effect was observed up to 24 h after oral administration to diabetic rats, unlike the one obtained for uncoated insulin-loaded SLN (Figure 8). Therefore, the solid matrix of SLN protected insulin against degradation in the gastrointestinal tract, and the additional chitosan coating enhanced its intestinal absorption, which may contribute to the development of an alternative route for the conventional subcutaneous administration of insulin and the improvement of diabetes management [115]. Within the same research group, it was also observed that chitosan coating provides stealth properties that reduce macrophage phagocytosis [17,116]. 

Some biological macromolecules such as nucleic acids cannot diffuse through the cell membrane due to their highly anionic nature and hydrophilic properties; however, their association with cationic lipid nanoparticles might enhance their intracellular delivery [117,118,119]. In a study, Cheng and his colleagues studied the modification of the composition of lipid nanoparticles by adding different ionizable cationic lipids, called Selective Organ Targeting (SORT) molecules, to evaluate if these altered nanoparticles could improve specific organ delivery without compromising the efficiency of the drug delivery. By administering SORT LNPs in mice by intravenous administration, elevated levels of mRNA delivery and tissue-specific gene editing were achieved. Diverse types of SORT molecules were evaluated, such as 1,2-dioleoyl-3-trimethylammonium-propane (DOTAP) and 1,2-dioleoyl-sn-glycero-3-phosphate (18PA) on diverse types of lipid nanoparticles. It was observed that the organ-selective transfection of mRNA delivery in mice by SORT LNPs in the lung, spleen, and liver were enhanced, and were also successfully produced in relevant levels, for therapeutic proteins such as IL-10, EPO, and Klotho. Additionally, the co-delivery of Cas9 mRNA and sgPCSK9 by SORT LNPs also enables a complete knockout of serum and protein levels of PCSK9, a therapeutically attractive target for the treatment of cardiovascular diseases [120]. Later, Wang et al. developed a protocol for the preparation of multiple classes of SORT LNPs by pipette, vortex, and microfluidic mixing methods as well physical characterization, and in vitro/in vivo mRNA delivery evaluation. These SORT LNPs might be applied to multiple classes of lipid nanoparticle systems for therapeutic nucleic acid delivery and also enable the development of protein replacement and genetic medicines in the desired tissues [121]. Finally, Algarni et al. studied the effect of cationic lipid nanoparticles on the intracellular delivery of a plasmid DNA (pDNA) to an organ-selective gene expression. In this work, DLin-MC3-DMA, DLin-KC2-DMA, or DODAP (1,2-dioleoyl-3-trimethylammonium-propane) were used as the ionizable cationic lipid component of the lipid nanoparticle in the study of the pDNA transfection efficiency in different mouse organs after intramuscular and intravenous administration. In the end, it was found that lipid nanoparticles combined with DLin-KC2-DMA revealed a significantly higher in vivo protein production in some organs to the detriment of others, in this case higher in the spleen than in the liver. Therefore, when nanoparticles have similar physiochemical properties, the transfection efficiency is mainly promoted due to the ionizable lipid nanoparticle structure rather than to the biodistribution of the lipid nanoparticles [118].

The possibility to modify the surface of both SLNs and NLCs is also relevant as a valuable tool to overcome the lack of targeted cancer therapies. One example of that is the modification of a drug delivery system with vascular endothelial growth factor (VEGF), since its receptors (VEGFRs) are overexpressed on the surface of a broad variety of tumor cells and vasculature. Therefore, targeting the chemotherapeutic agents to the VEGFR-overexpressed tumoral cells and tumoral neovascular endothelial cells in vitro and in vivo increase the treatment selectivity. Liu et al. designed and prepared an antibody-modified docetaxel-loaded targeted NLC (tNLC) with the polymer DSPE-PEG-NH2 as linker. Cellular toxicity of tNLC against three human cell lines and one murine malignant melanoma was found to be higher than non-encapsulated docetaxel and non-targeted NLC (nNLC). A higher tolerance and antitumor efficacy in a murine model bearing B16 was shown for tNLC compared to docetaxel or nNLC. Thus, tNLC applied as a drug delivery system targeted to bind specifically to VEGFR-2 via anti-VEGFR-2 antibody promotes the biodistribution, and anti-tumoral effect of docetaxel, due to a specific binding and increased accumulation of the drug in both tumoral cells and tumoral microvasculature [122]. In another work, Nawaz et al. delivered VEGF-A mRNA via lipid nanoparticles to ischemic tissues and studied their uptake kinetics and how the transport of exogenous lipid nanoparticles-mRNAs between cells is compared with that realized by cells vehicles, the extracellular vesicles. The study revealed that the cellular uptake of lipid nanoparticles and their mRNA molecules occurs quickly, as well as the beginning of the translation of exogenously delivered mRNA. Moreover, a fraction of internalized overexpressed VEGF-A mRNA is secreted via extracellular vesicles. Finally, in vivo injections of VEGF-A mRNA (via extracellular vesicles or lipid nanoparticles) into mouse hearts resulted in locally produced VEGF-A protein without enforcement to liver and circulation. So, in general, these results showed that lipid nanoparticles transform extracellular vesicles as functional extensions to share out therapeutic mRNA between cells, in which extracellular vesicles provide this mRNA in a different way than lipid nanoparticles [123]. Zha et al. also have also taken advantage of the potential of the delivery of VEGF-A mRNA by ionizable lipid nanoparticles, but in this case to increase the absorption and half-life of the protein macromolecule with the aim of the promotion of the healing of the diabetic wound. In the end, the nanoparticles revealed a promotion of endothelial cell proliferation exhibited a high VEGF-A protein expression in vitro and in vivo associated with a good mRNA delivery. In a diabetic mice model treated with the loaded VEGF-A mRNA lipid nanoparticles was found, after 14 days, successful treatment of wounds with an average wound area of 2.4% while in the control group (treated with the PBS) it was 21.4%. Therefore, the administration of these lipid nanoparticles was revealed to be a potential strategy for protein replacement therapy to improve chronic diabetic wound healing, once it promotes the bioavailability of VEGF-A mRNA and the protein expression [124].

SLN and NLC surfaces can also be modified with antibodies that target receptors overexpressed in cancer cells [125]. Di Filippo et al. developed an NLC-loading docetaxel modified with Bevacizumab to target the angiogenic factor VEGF in cancer cells of glioblastoma multiforme cancer. The nanosystem was able to promote cell death by apoptosis electively in glioblastoma cells while not having a cytotoxic effect in healthy immune cells. Moreover, NLCs were shown to improve brain drug delivery, mostly due to the increased BBB permeation. Finally, the bevacizumab-conjugated NLC-loading docetaxel were able to reduce up to 70% of an orthotopic preclinical tumor model in rat, whereas free docetaxel did not [126].

Another example is the importance of FA for cancer cells. Such cells need an increased quantity of FA for their DNA synthesis and division since the activity of the folic acid receptor in the membrane of cancer cells is higher than in normal cells, so this molecule has been linked to other molecules to promote their uptake by cancer cells [127] and using FA as a targeting moiety, SLNs and NLCs may easily undergo receptor-mediated phagocytosis [128]. A recent study using ultrasmall (about 50 nm) NLCs with FA modification (FA-NLC) to load docetaxel not only improved targeting and significantly decreased the volume of the tumor in a mouse model, but also reduced the side effects of the drug. The intracellular uptake was evaluated in HeLa cells (cervical cancer cells) which were incubated subcutaneously in the flank region of the mice that highly expressed the folate receptor. The commercial version of docetaxel reduced the bodyweight of mice by roughly 1 g, representing a loss of almost 5% (starting weight of mice between 22 and 23 g), whereas the docetaxel loaded in NLCs showed no decrease. This showed that NLCs reduced the side effects of the drug by preventing its interaction with other tissues, other than the HeLa tumor xenograft mice, as well as allowing better permeation and higher accumulation in the tumor. Furthermore, these nanoparticles exhibited high colloidal stability and satisfactory drug loading efficiency [129]. 

Another well-known strategy is surface modification with PEG as it prevents its capture by the reticuloendothelial system [129]. Besides that, surface nanoparticle modification with PEG has been shown to increase their internalization efficiency and the cross-cell membrane, probably because PEG has good solubility for both polar and nonpolar solvents and therefore in cell membranes [86]. Thus, surface modification with PEG, PEGylated SLN, is a good strategy not only to improve the protection of the nanoparticles but also to improve their therapeutic efficacy by enhancing cellular uptake. 

Other studies using cell penetrating peptides showed similar results, with an enhanced cytotoxic effect on cancer cells and uptake by cells, when compared with either free drug or NLC formulations without “active” targeting [130,131,132]. Multifunctional NLC formulations are also described in the literature. In a described study, its co-loaded quantum dots (CdTe/CdS/ZnS) and paclitaxel into NLC as a parenteral multifunctional delivery system. This system showed the ability to target and detect H22 tumors in mice. The mice were subjected to near-infrared fluorescence imaging to follow the treatment evolution, resulting in an efficient theragnostic model [133]. Other authors used another approach to the concept of multifunctional nanoparticles, by using as an active targeting a synthetic luteinizing hormone-releasing hormone decapeptide onto NLC surface, to deliver directly to non-small cell lung carcinoma in mice, both paclitaxel and small interfering RNAs to suppress tyrosine kinase inhibitors acting on the epidermal growth factor receptors (EGFR-TK). The results showed this multifunctional model was more efficient in cancer therapeutics when compared to the free drug, one inhibitor of EGFR-TK, and non-targeted NLC [134]. 

The dermal use of lipid nanoparticles shows many benefits besides the chemical protection of the loaded drugs, such as improving the skin penetration and retention of labile molecules that are not easily delivered in a traditional semi-solid formulation, allowing local and controlled release. These advantages are due to the physiological lipid nature of nanocarriers that promotes the interaction with the subcutaneous tissue creating its lipid rearrangement. These effects are also improved by the penetration enhancer effect of the surfactant in the formulations [135,136]. In this sense, NLCs and SLNs can be engineered to formulate hydrogels to enhance the skin permeation of several drugs. A study developed by Sütő et al. described a NLC based gel for the topical delivery of ibuprofen, for the treatment of osteoarthritis and other musculoskeletal diseases. Ibuprofen is widely used due to its potent anti-inflammatory properties since its skin permeation ability is well known. However, by encapsulating the nonsteroidal anti-inflammatory drug it was possible to increase by nearly 2-fold the penetration of ibuprofen when compared to a “traditional” gel, in addition to the fact that this system presented an entrapment efficiency of 98.51% [137]. Similarly, Puglia et al. showed that NLC-loading benzocaine and lidocaine had a longer release profile, thus increasing the exposition of the anesthetic effect and therapeutic efficacy [138]. Zhao and their colleagues reached a similar conclusion using a modified cationic NLCs for the transdermal application of lidocaine. They engineered a tocopheryl PEG 1000 succinate-modified cationic NLCs that showed a prolonged and efficient local anesthetic therapy [139]. Viegas et al. also developed a multifunctional NLC to co-deliver tacrolimus and siRNA, to treat psoriasis by tackling the TNF-α (one of most cytokines expressed in psoriasis), through topical administration in an animal model. This system showed a controlled release of tacrolimus and good outcomes in permeation and retention profiles in the skin. It was observed a 7-fold reduction of TNF-α expression and a synergic effect between tacrolimus and TNF-α siRNA, successfully treated psoriasis in the in vivo model [140]. 

To emphasize the benefits of a matrix based on a mixture of solid and liquid lipids to controlled delivery, Esposito et al. produced and characterized tristearin SLNs and NLCs made of tristearin with caprylic triglyceride loaded with progesterone to enhance the skin uptake of the drug. The results showed that both SLNs and NLCs had good physical and chemical stability without agglomeration phenomena over 6 months from the production. However, NLCs revealed a slower drug release than SLNs. Moreover, human in vivo studies showed a decrease in progesterone concentration in the stratum corneum after 6 h, revealing an interaction between nanoparticles and skin lipids [141].

Another benefit that can be taken from these systems for skin application is concerning skin hydration by an occlusive film formation and avoidance of water loss by evaporation, or reinforcement of the skin lipid film barrier by nanoparticle adhesion to the stratum corneum [136,142]. Tichota et al. developed an NLC system, using argan oil as a liquid lipid due to its moisturizing properties, as a promising strategy for dermal delivery to improve skin hydration, once a synergistic effect on the skin hydration was achieved (argan oil hydration and occlusion by NLC) [143]. Another study performed by Souza et al. proposed the development of beeswax-based nanoparticles for the dermatological and cosmetic use of age-cream base formulation aiming to recover damaged skin barrier function. After 28 days of applying the semi-solid beeswax SLN formulation, the authors observed a significant decrease in the water loss by evaporation and an increase in stratum corneum water content, showing its potential use in skin diseases treatment and recovering of damaged skin barrier function [41]. 

However, topical drug administration still faces many challenges, such as controlling and determining the amount of drug that reaches different skin layers. Lipid nanoparticles have shown promising results in the last few years to bypass these challenges, by enhancing percutaneous absorption and specific drug targeting [61]. Štecová et al. developed SLNs made with Precirol, and NLCs made with Precirol and Oleic Acid or Mygliol as lipid nanocarriers, to the delivery of cyproterone acetate (CPA) 0.05%, for the treatment of acne vulgaris. It analyzed the skin penetration and permeation of CPA in both nanocarriers. In SLNs as in NLCs, a high encapsulation efficiency was achieved. However, in the human skin strips where SLNs were used, the penetration of CPA increased 4-fold when compared to the cream, while in NLCs the increase was only 2-fold. Regarding skin permeation, the values obtained were 1.30 ± 0.27% with SLN, 0.51 ± 0.007% with NLC-O (with oleic acid), and 0.67 ± 0.05% with NLC-M, (with Mygliol). Even though the values of skin permeation by CPA are low, they still represent a 4-fold increase for SLNs when compared to skin permeation when only the cream was applied. Hence, lipid nanoparticles enhanced skin penetration and permeation when compared with the cream formulation and can be a promising delivery system for topical delivery in the future [144]. 

As mentioned before, the study of lipid nanoparticles has also increased in recent years as carriers and protection devices for mRNA-based vaccines, which have the potential to treat several diseases that presently are considered uncurable. The works performed so far show lipids as a primary driver. With the emergence of the COVID-19 pandemic, numerous studies carried out by different biotech companies emerged, trying to develop an mRNA-based vaccine to confer immunity against COVID-19 [83,145]. One of these studies, made by Moderna, evaluated 30 novel lipid nanoparticles, with a different SORT molecule, in terms of stability, target delivery, immunogenicity, and adverse effects. Lipid nanoparticles were confirmed as effective adjuvants for protein subunit vaccines; however, no correlation has yet been established between the adjuvant mechanism and the potency of the immune response [145]. 

## 6. Toxicity Concerns

In every study on formulation development, the evaluation of the biocompatibility of SLNs and NLCs is crucial. The encapsulation of the drugs in SLN and NLC and its targeting of specific cells is expected to increase selectivity, and thus decrease toxicity problems [146]. This is achieved by either enhancing nanoparticle-cell contact (e.g., targeting moieties, and positive charge surface) or overcoming drug efflux transporters (e.g., p-glycoprotein). However, for both SLN and NLC, the lipidic content should not impact cell viability, but surfactants used in formulations may present cytotoxicity [147]. The most efficient way to compare cell viability is the half maximal inhibitory concentration value. IC_50_, however, it is not evaluated in every toxicity study. In the literature, cell sensitivity to different lipidic formulations is usually expressed as cell viability and it shows that cells rarely tolerate higher concentrations of lipid carriers. Interestingly, few studies suggest that cancer cell lines may be more sensitive to SLNs and NLCs than non-cancer cell lines; however, more studies are needed to conclude [148,149,150,151].

Another concern regarding SLNs and NLCs is that even if the lipids and other excipients are GRAS, a cytotoxicity study should still be done to confirm if they have no cytotoxic effect on the cells, for example, after being digested. Even though they are proven safe and can be used for oral and topical administration, this assumption is not valid with parenteral delivery since surfactants can also activate the immune system [146]. 

One study that supported the importance of the evaluation of bio-based ingredients’ cytotoxicity and cytotoxic effect after in vitro digestion was the work done by Gonçalves and his colleagues. This study concluded that NLCs constituted of medium-chain triglycerides oil, a GRAS lipid, showed cytotoxic effect over the cell lines after digestion and, consequently, reduced cell viability, due to the products resulting from that lipid digestion [105]. Therefore, more studies should be performed to evaluate the toxicity of SLNs and NLCs after administration, even if the lipids are recognized as safe.

Even if the overall formulation of SLNs and NLCs is well-tolerated, its components alone (lipid core material and surfactant) are rarely accessed for cell viability. Different core materials, such as Compritol^®^ 888 ATO and trimyristate, have different degradation rates since longer-chain lipids take longer to break down due to lipases activity, resulting in a slightly higher impact on cell viability by Compritol^®^ SLNs than trimyristate SLN. Other studies revealed that depending on the cell line, the same core material, such as stearic acid, has more toxicity toward primary macrophages than HL-60 macrophages [152,153]. Despite stearic acid being a widely used lipid in SLN and NLC formulations, its cytotoxic effects have been poorly studied. SLN and NLC formulations may form aggregates, suffer structural changes (e.g., desorption of surfactants), or even adsorption of proteins presents in cell culture media. Evaluating the isolated core materials in the same amount and ratios used in the formulations may contribute to clarifying the observed effects. Thus, the surfactant type, surface coverage, and bound to lipid core are fundamental to the performance of SLN and NLC formulation in cell cultures.

Regarding the toxicity of the used solvents, most of the preparation methods aim to decrease or avoid the use of potentially toxic organic solvents and other toxic additives, which are an advantage of these types of lipid formulations. Moreover, in cases where an organic solvent is necessary to dissolve the lipid, there is later evaporation of the organic solution. This is also valid for large scale production. Therefore, this is not an issue during the formulation and delivery of SLN and NLC [29,154,155].

The surfactants commonly used are non-toxic; however, SLN formulations with the same lipid core, but different surfactants, show slight differences in cell viability. This may be due to the interaction between surfactant and particle surface, resulting in a decreased bioavailability to interact with living cells and induce toxicity [148]. Various studies have been carried out to identify if the type of surfactant used and its concentration can influence the cytotoxicity of the cell. However, even though there have been some slight changes in cell-nanocarrier interaction, for lipid nanoparticles made with the same lipids and different surfactants, no conclusive evidence of cytotoxicity has been observed or linked to the surfactant until today. There has been some evidence that the type of surfactant is more important when referring to cationic SLNs and NLCs. However, no conclusion has been reached since these nanoparticles alone have higher levels of cytotoxicity when compared to anionic-charged SLNs and NLCs since most cancer cells have negatively charged surface moieties that attract positive charges [23,156]. For instance, the cationic amphiphilic surfactant cetyltrimethylammonium bromide used in the formulation of NLCs exhibits cytotoxicity because it can interact with the neutrophils cell membrane, which disturbs its integrity, therefore promoting calcium influx. This elevation of intracellular calcium will induce cell damage by oxidative stress and consequently, cell death [157,158]. 

In another study, NLCs prepared with a mixture of other surfactants, such as polysorbate 80, poloxamer 188 and Tween^®^ 80 showed good biocompatibility and low cytotoxicity [159]. Although it has been found that the use of a blend of surfactants can improve the stability of NLC by increasing the charge of the nanoparticles, which results in greater electrostatic repulsion at the interface with the medium, and therefore improving the physical stability of the colloidal system, these blends may increase the risk of toxicity of the formulation [154,160].

Another concern is the extra material used to produce NLCs, the oil component. Theoretically, it should be incorporated inside the core to increase the stability of the formulation by inhibiting polymorphic phase changes. Moreover, it has been reported a phenomenon where the oil component forms a film attached to the particle surface, being more available to interact with cells but it is yet unknown whether the oil stays in the particle surface or may suffer desorption as surfactants. However, due to limited data on toxicity comparing the two systems, it is difficult to conclude which is less toxic, as no apparent difference was found [161,162].

One toxic effect associated with lipid nanoparticles, when it comes to skin administration, is their potential to increase the phototoxicity of UV radiation in the dermal fibroblasts. Brugè et al. revealed that possibility [163]. Furthermore, lipid nanoparticles have also been associated with the formation of radical oxygen species (ROS) at the mitochondrial level due to interruptions of the respiratory cycle. Later, the same authors formulated five types of blank NLC made of the most common lipids and surfactants used in different studies to study the effect of these nanoparticles in the fibroblasts regarding ROS production with and without UV radiation. They observed that for all NLCs, except one, mild levels of ROS were detected in the cell lines not exposed to the UV light, with an increase of up to 98% when compared to the control. When comparing those with the cell lines exposed to UV radiation, the NLC cytotoxicity of most was exponentially enhanced, in some cases increasing the number of dead cells by 468% [164]. Hence, more cytotoxic studies should be made regarding the formation of ROS, especially when it comes to topical administration. 

Another concern for SLNs and NLCs is DNA damage by base oxidation, strand breaks, or even DNA repair disruption. Studies performed so far showed that SLNs and NLCs do not cause genotoxicity. The analysis of gel electrophoresis showed the absence of DNA fractionation in the A549 cell line treated with SLN formulations. This was confirmed by other studies using the more sensitive comet assay (single cell gel electrophoresis assay). In this method, the comet tail is compared to the comet head (cell nuclei) by measuring fluorescence intensity. The absence of fluorescence or no increase in fluorescence in the comet tail indicates a minimal risk of genotoxicity. In the few cases where the drug-loaded SLNs caused a slight increase in comet tail intensity, a similar effect was observed with the free drug. However, as the materials used in SLN and NLC formulations dictate the cell tolerance to the formulation, more studies are needed with different formulations [163,165,166]. 

## 7. Conclusions

Overall, SLNs and NLCs have demonstrated great potential as drug delivery systems for therapeutic applications. While both systems have similar applications, NLCs are preferred due to their greater stability and drug loading capacity. These systems offer a highly flexible and controlled release profile, which is leading to increased interest and impact in various fields, including medicine, gene editing, food, and cosmetics. Numerous studies in the past decade have shown that these lipid-based drug delivery systems are low in toxicity at therapeutic quantities due to their composition in physiological lipids and avoidance of organic solvents. They also allow increased drug protection, load both lipophilic and hydrophilic drugs, a high absorption rate, biocompatibility, and biodegradability. A targeted drug delivery and enhanced drug permeation are also an advantage. However, more research is needed to reach a more reliable conclusion on cytotoxicity to ensure their clinical success. Despite the challenges, it is expected that the development of these nanocarriers will continue to evolve in the coming years, leading to the development of third-generation lipid nanocarriers that can address all the pitfalls of lipid nanoparticles, offering superior therapeutic benefits. This exciting prospect underscores the potential of lipid-based drug delivery systems as a valuable tool for drug delivery in various therapeutic applications.

## Figures and Tables

**Table 1:** Chemical classification, source, and function of the compounds used to produce SLNs and NLCs.

Compound	Classification	Source	Function
1-Tetradecanol (myristyl alcohol)	Straight chain saturated fatty alcohol	*Myristica fragrans*	Solid lipid
Beeswax	Wax ester	Honey bees (*Apis mellifera*)	Solid lipid
Caprylic/capric triglyceride	Triglyceride	Coconut oil	Liquid lipid
Castor oil	Fatty acid composed	Castor beans	Liquid lipid
Cetyl palmitate	Wax ester	Stony corals, *Psidium guajava*	Solid lipid
Cholesteryl myristate	Cholesterol ester	*Trachyrhamphus serratus*	Solid lipid
Cholesterol	Modified steroid	Animal, vegetable fat	Solid lipid
Compritol^®^ 888 ATO	Mixture of mono-, di- and triglycerides of behenic acid (C22)	-	Surfactant
1,2-dioleoyl-3-dimethylammonium propane (DODAP)	Ionizable cationic lipid	-	Solid lipid
Dipalmitoylphosphatidylcholine (DPPC)	Phospholipid	Pulmonary surfactant	Solid lipid
1,2-distearoyl-sn-glycero-3-phosphoethanolamine (DSPE)	Amine phospholipid	*Escherichia coli*	Solid lipid
Gelucire^®^ 50/13	Mixture of fatty acids (C16 and C18), esters of glycerol, PEG esters and free PEG	-	Surfactant
Glyceryl monostearate	Glycerol ester of a saturated fatty acid	*Aristolochia cucurbitifolia, Lobelia longisepala*	Surfactant
Labrafac™ CC	Mixture of medium chain triglycerides, mainly from caprylic (C8) and capric (C10) acids	-	Liquid lipid
Lecithin	Mixture of phospholipids in oil	Soybean, egg	Surfactant
Miglyol^®^ 812 N	Glycerol triester of caprylic and capric acid (triglyceride esters)	Coconut, palm kernel oil	Liquid lipid
Myristylmyristate	Tetradecanoate ester	Coconut, palm kernel oil	Solid lipid
Oleic acid	Middle chain triglyceride	Olive oil	Liquid lipid
Palmitic acid	Saturated fatty acid	Palm oil	Solid lipid
Phosphatidylcholine	Phospholipid	Soybeans, eggs	Solid lipid
Poloxamer 407/Pluronic^®^ F-127	Triblock copolymer	-	Surfactant
Precirol^®^ ATO-5	Mixtures of diesters of glycerin and stearic acid	-	Solid lipid
Polyvinylalcohol (PVA)	Synthetic polymer of vinyl alcohol	-	Surfactant
Sodium lauryl sulfate (SLS)	Ethoxylated lauryl alcohol	Coconut, palm kernel oil	Surfactant
Squalene	Triterpenoid	Olive, wheat germ, and rice bran oils	Liquid lipid
Steric acid	Saturated fatty acid	Animal, vegetable fat	Solid lipid
Tricaprin	Triglyceride	Milkfat, palm kernel oil, and coconut oil	Solid lipid
Tripalmitin	Triglyceride	*Lysiphlebia japonica, Tagetes erecta*	Solid lipid
Tristearin	Triglyceride	*Lysiphlebia japonica, Sciadopitys verticillata*	Solid lipid
Tween^®^	Mixture of sorbitol, ethylene oxide, and oleic acid	-	Surfactant

**Table 2:** Examples of studies performed using SLN. The nanoparticles components and features, loaded drug, production method, and therapeutic purpose are debriefed.

Solid–Lipid	Surfactant	Drug	Production Method	Therapeutic Purpose	Delivery Route	Characteristics	Ref
Gelucire^®^ 50/13	Tween^®^ 85	Grapeseed-derived proanthocyanidins	Melt Emulsification Technique	Chronic Respiratory Diseases	Spray Instillation	Size: 243 ± 24 nm PdI: 0.41 Zeta: −14.5 ± 1.0 mV EE: NA	[36]
Palmitic Acid/Cholesteryl Myristate (68,5/31,5%) (*w*/*w*)	Sodium Lauryl Sulfate (SLS)	Rifampicin	Melt Emulsification Technique	Tuberculosis	NA	Size: 400 ± 20 nm PdI: 0.43 ± 0.09 Zeta: −35.3 ± 0.29 mV EE: 56.48% (*w*/*w*)	[37]
Compritol 888 ATO, cholesterol, and Tf-PEG-OA	1% Polyvinylalcohol (PVA)	Paclitaxel (PTX)	Solvent Evaporation Method	Leukemia	NA	Size: 176 nm PdI: NA Zeta: −22.5 ± 1.56 mV EE: 92.5 ± 1.35%	[38]
Tripalmitin/Hydrogenated soybean phosphatidylcholine (HSPC) (80/20%) (*w*/*w*)	Polyethylene glycol monostearate (PGM)	Apomorphine	NA	Parkinson’s Disease	Oral	Size: 63.20 ± 0.98 nm PdI: 0.31 ± 0.02 Zeta: 7.3 ± 0.25 mV EE: NA	[39]
Compritol^®^ 888 ATO	Tween^®^ 80	Quercetin	NA	Alzheimer’s Disease	Oral	Size: 0.42 to 4.62 µm PdI: NA Zeta: −23.6 to −5.13 mV EE: 85.7%	[40]
Beeswax	Tween^®^ 80 Poloxamer 407	NA	Hot melt microemulsion	Skin Hydration	Topical	Size: 95.72 ± 9.63 nm PdI: 0.323 ± 0.03 Zeta: −9.85 ± 0.57 mV EE: NA	[41]
Stearic Acid	Poloxamer 407 Soybean Phosphatidylcholine	Resveratrol	Sonication	Anti-tumoral	Topical	Size:155.50 ± 0.26 nm PdI: 0.140 ± 0.02 Zeta: −2.60 ± 1.27 mV EE: NA	[42]
Poly Lactic-co-Glycolic Acid (PLGA)	1% polyoxyethylenepolyoxypropylene	Apigenin	Nanoprecipitation	Cosmetic	Topical	Size: 102.19 ± 0.002 nm PdI: 0.258 Zeta: 12.1 ± 0.0 mV EE: 87.2 ± 0.005	[43]
Tricaprin	Cetyl Palmitate, Tween^®^ 60 Tego Care 450 Amphisol K, 1-Tetradecanol	Resveratrol	Hot melt homogenization	Cosmetic	Topical	Size: 102.190 ± 0.002 nm PdI: 0.258 Zeta: 12.1 ± 0.0 mV EE: 52.45%	[44]

**Table 3:** Examples of studies performed with NLC. The nanoparticles’ components and features, drug loading, production method and therapeutic purpose are debriefed.

Solid Lipid	Liquid Lipid	Surfactant	Drug	Production Method	Therapeutic Purpose	Delivery Route	Characteristics	Ref
Stearic acid	Oleic acid	Soya Lecithin Glyceryl Monostearate	Docetaxel (DTX)	Modified film ultrasonication–dispersion method	Murine Malignant Melanoma	Parenteral	Size: 203.67 ± 4.15 nm PdI: NA Zeta: −31.17 ± 2.20 mV EE: 89.39 ± 0.99%	[49]
Precirol^®^ ATO-5	Squalene	Myverol	Lovastatin	Hot melt homogenization	Cholesterol	Oral	Size: 278.8 ± 0.6 nm PdI: ≤0.25 Zeta: −32.4 ± 0.4 mV EE: 83.8 ± 2.5	[50]
Comprito^®^ 888 ATO	Miglyol 812N	Lecithin	Vinpocetin (VIN)	High-pressure homogenization	Brain Disorders	Oral	Size: 177 ± 5.4 nm PdI: NA Zeta: −24.7 ± 1.4 mV EE: 95.3 ± 1.4	[51]
Precirol^®^ ATO-5	Oleic Acid	Tween^®^ 80	1-carbaldehyde-3,4-dimethoxyxanthone (LEM2)	Ultrasonication	Melanoma	Topical	Size: 219.67 ± 5.26 nm PdI: ≤0.3 Zeta: −24.88 ± 1.78 mV EE: 72%	[52]
Cetyl Palmitate	Miglyol 812N	Tween^®^ 60	Curcumin	Modified hot homogenization	Brain Disorders	Oral/Intravenous	Size: 183 ± 12 nm PdI: 0.13 ± 0.01 Zeta: −21 ± 2 mV EE: 82 ± 15%	[53]
Glyceryl Tribehenate	Oleic acid	P 407	Raloxifene hydrochloride (RLX)	Hot homogenization	Osteoporosis	Oral	Size: 120 ± 3 nm PdI: 0.293 Zeta: 14.4 ± 0.5 mV EE: 91.71	[54]
Precirol ATO-5	Miglyol 812N	Tween^®^ 80	Rifapentine (RPT)	Hot ultra-sonication	Tuberculosis	Oral/Pulmonary	Size: 242 ± 9 nm PdI: 0.17 ± 0.01 Zeta: −22 ± 2 mV EE:	[55]
Glycerol monostearate (GMS)	Medium chain triglyceride (MCT)	Poloxamer 188 Soybean lecithin	Amoitone B	Emulsion-evaporation and low temperature-solidification	Tumor Therapy	NA	Size: 241.2 ± 4.4 nm PDI: NA Zeta: 18.4 ± 0.2 mV EE: 71.5 ± 1.1%	[56]
Myristyl Myristate	Crodamolt GTCC-LQ	Pluronic F128	Metvan	Sonication	Bone Cancer	NA	Size: 230.8 ± 3.1 nm PdI: 0.235 ± 0.010 Zeta: −7.9 ± 0.8 mV EE: 77.6 ± 4.8%	[57]
Stearic acid or beeswax	Carvacrol	Kolliphor188^®^	Carvacrol	Warm microemulsion oil in water (*o*/*w*)	Leishmaniasis	Parenteral	Size: 98 ± 0.80 nm PdI: 0.166 ± 0.04 Zeta: −25 ± 5 mV	[58]

**Table 4:** Comparison between SLN and NLC. Advantages and disadvantages.

	SLN	NLC
Lipids	Use of physiological lipids; however, there is a lower stability comparatively with other materials	
Solvents	Absence of organic solvents	
Application	Application in different industries (food, cosmetic, pharmaceutical)	
Bioavailability	Improved bioavailability of drugs	
Drugs loaded	Loads both lipophilic and hydrophilic drugs; however, has difficulty in loading therapeutic proteins	
Drug delivery	Targeted drug delivery and enhanced drug permeation	
Scale-up	Cheaper and easier to scale up than polymeric nanoparticles	
Protection	Protection of drug molecules from enzymatic activity, harsh pH, and moisture	
Cytotoxicity	Cytotoxicity concerns due to the nature and concentration of matrix lipids	
Drug loading capacity	Limited drug loading capacity	Improved drug loading capacity
Controlled drug release profile	Difficulty in adjusting the drug release profile	Better controlled drug release profile
Polymorphic transitions	Prone to polymorphic transitions	No polymorphic transition takes place
Release during storage	Unwanted drug release during storage	Minimal drug release during storage
Physical stability	Possible particle aggregation or fusion during storage	Better physical stability during storage
Water content	High water content	Low water content

## Data Availability

Not applicable.

## Abbreviations/Nomenclature

18PA	1,2-dioleoyl-sn-glycero-3-phosphate
BBB	Blood brain barrier
CLA	Conjugated linoleic acid
CPA	Cyproterone acetate
DODAP	1,2-dioleoyl-3-dimethylammonium-propane
DOTAP	1,2-dioleoyl-3-trimethylammonium-propane
DPPC	Dipalmitoylphosphatidylcholine
DSPE	1,2-distearoyl-sn-glycero-3-phosphoethanolamine
EGFR-TK	Epidermal growth factor receptors
FA	Folic acid
GRAS	Generally recognized as safe
HPH	High-pressure homogenization
HSPC	Hydrogenated soybean phosphatidylcholine
NLC	Nanostructured lipid carriers
nNLC	Non-targeted nanostructured lipid carriers
PdI	Polydispersity index
pDNA	Plasmid DNA
PEG	Polyethylene glycol
PLGA	Poly Lac-tic-co-Glycolic Acid
PVA	Polyvinylalcohol
RH	Relative Humidity
ROS	Radical oxygen species
SLN	Solid–lipid nanoparticles
SLS	Sodium lauryl sulfate
SORT	Selective Organ Targeting
SORT LNPs	Selective organ targeting lipid nanoparticles
tNLC	Target nanostructured lipid carriers
VEGF	Vascular endothelial growth factor
VEGFR	Vascular endothelial growth factor receptor
